# A Recast Framework for Welfare Deservingness Perceptions

**DOI:** 10.1007/s11205-021-02774-9

**Published:** 2021-08-20

**Authors:** Carlo Michael Knotz, Mia Katharina Gandenberger, Flavia Fossati, Giuliano Bonoli

**Affiliations:** 1 Department of Media and Social Sciences (IMS), University of Stavanger, Elise Ottesen-Jensens Hus, Kjell Arholms Gate 37, 4021 Stavanger, Norway; 2 Swiss Graduate School of Public Administration (IDHEAP), University of Lausanne & NCCR - on the move, Quartier UNIL-Mouline, Bâtiment IDHEAP, 1015 Lausanne, Switzerland; 3 Swiss Graduate School of Public Administration (IDHEAP), University of Lausanne, NCCR - on the move & NCCR - LIVES, Quartier UNIL-Mouline, Bâtiment IDHEAP, 1015 Lausanne, Switzerland

**Keywords:** Deservingness, CARIN, Social solidarity, Welfare state, Vignette experiment

## Abstract

Many important societal debates revolve around questions of deservingness, especially when it comes to debates related to inequality and social protection. It is therefore unsurprising that a growing body of research spanning the social and political sciences is concerned with the determinants of deservingness perceptions. In this contribution, we engage with the currently central theoretical framework used in deservingness research and point out an important weakness: Partly ambiguous definitions of the framework’s central concepts, the criteria for perceived deservingness. We also highlight the negative consequences this has for empirical research, including notably varying and overlapping operationalizations and thereby a lacking comparability of results across studies. Our main contribution is a redefinition of the criteria for perceived deservingness and a demonstration of the empirical implications of using this new set of criteria via original vignette survey experiments conducted in Germany and the United States in 2019. Our results provide a clearer image of which criteria drive deservingness perceptions.

**Supplementary Information:**

The online version contains supplementary material available at 10.1007/s11205-021-02774-9.

## Introduction

The last decades have seen a significant reshaping of welfare state institutions in economically advanced democracies, and it has long been recognized that citizens’ attitudes have a strong influence on whether welfare state reforms are introduced and which shapes they take (e.g., Brooks & Manza, 2006). More specifically, it has been shown that citizens’ perceptions of the deservingness of the target groups of different social protection programs play a considerable role for policymaking: Target populations that are commonly seen in a positive light are less likely to be the targets of cutbacks and more likely to benefit from expansions than those who are seen in a negative light (Schneider & Ingram, 1993). An example is the variation in the extent to which countries impose tougher activation requirements on the unemployed: It is typically the young unemployed that are first subjected to such requirements, whereas older workers are more often exempted (Larsen, 2008a). Similarly, the negative public image of benefit claimants with immigrant or minority backgrounds (Alesina et al., 2018; Gilens, 1996), and the recent emergence of immigration as a highly salient political issue (Kriesi et al., 2012), can explain why countries have started to curtail immigrants’ access to social protection, and why such reforms are championed by both populist right-wing and mainstream parties (Careja et al., 2016). Given the importance of public perceptions of deservingness for policymaking, it is crucial to understand how these perceptions are formed and why some groups are seen as more deserving than others.

Precisely this has motivated the development of microlevel approaches to welfare deservingness perceptions. In a highly influential article, van Oorschot (2000) developed a framework to explain the variation in the perceived deservingness of different claimant groups. His framework predicts that persons or groups should be seen as more deserving the more they fulfil the following five criteria: (1) they experience hardship due to factors outside of their control; (2) they display a grateful and docile attitude; (3) they have contributed to others in the past or are currently trying to do so; (4) they are similar in their social identity; and (5) they are really in need of help. Together, these five criteria—control, attitude, reciprocity, identity, and need—form the CARIN criteria for perceived deservingness. More recent research has also provided insights into the deeper psychological mechanisms behind deservingness perceptions, and suggests that these perceptions are driven by a cognitive “cheater-detection” mechanism that likely originated in early human hunter-gatherer societies (Aarøe & Petersen, 2014; Petersen, 2012, 2015; Petersen et al., 2012; see also Bowles & Gintis, 2000; Cosmides et al., 2010).

Despite the undeniable contributions recent research on deservingness perceptions has made, this literature suffers from one important but so far unresolved problem: the central theoretical concepts, the criteria for perceived deservingness, are in key respects ambiguously defined (as also noted by others before; see e.g., Reeskens & van der Meer, 2019, p. 172). For one, and as we illustrate in more detail below, there are overlaps between the definitions of some criteria, specifically the criteria of attitude and reciprocity. Second, some of the criteria are defined in a way that they encompass several different aspects that should arguably instead be captured by separate criteria. In more technical terms, the current conceptualization of deservingness criteria suffers from what Gerring (1999) called insufficient “differentiation” and “internal coherence”.

This creates obstacles for research. Generally speaking, concepts are the vocabulary we use to build theories (cf. Gerring, 1999), compare findings, and cumulatively build insights. But all this obviously becomes difficult if the conceptual vocabulary is ambiguous and if some words in this vocabulary can have different or overlapping meanings. More concretely, ambiguous concepts create problems in empirical analyses. First, ambiguously defined concepts are difficult to operationalize consistently across empirical analyses. There is therefore a risk that operationalizations vary between different studies, making it hard to directly compare their findings and cumulatively generate insights. Second, results also become more difficult to interpret when the underlying concepts are ambiguous.

We accordingly believe that much could be gained from a redefinition of the criteria for perceived deservingness, and this is what we contribute here. In the next section, we provide a more in-depth assessment of how the criteria are currently defined and used, and how this has affected deservingness research. In the third section, we develop our new set of criteria.

Following this, we demonstrate via three separate vignette survey experiments conducted in two different welfare states, Germany and the United States (US), that our new set of criteria leads to clearer insights about which criteria really matter for deservingness perceptions. Specifically, our evidence suggests that deservingness perceptions are driven by the following five criteria: the level of need (N), the extent to which one is seen as having a shared social identity (I), the level of control (C), current efforts to contribute (E), and past reciprocal behavior (R). Contrary to the predictions of the CARIN framework, the attitude criterion does not matter. In other words, our results suggest that CARIN should in fact be spelled NICER. We present our experiments in the fourth section and the results in the fifth section. We discuss the general implications of our findings but also some limitations of our study in the concluding section.

## Ambiguities in Definitions and Applications of Deservingness Criteria

Gerring (1999) developed a check list for social science concepts, which included among other requirements that concepts should be a) defined in a sufficiently narrow way so that they do not simultaneously capture different things (“internal coherence”) and b) should be clearly distinct from other related concepts (“differentiation”). The CARIN criteria as they are currently being used do not completely satisfy these two requirements. This concerns in particular the criteria of attitude and reciprocity.

In the case of the attitude criterion, internal coherence is a first issue. According to the original definition of the attitude criterion by van Oorschot (2000, p. 36), a grateful attitude can be expressed via essentially symbolic acts (e.g., a smile) but also concrete behavior (e.g., active job-search). Clearly, this definition is not illogical per se, since expending efforts such as looking for work when unemployed can be seen as a sign of gratefulness and docility. However, an essentially symbolic act such as a smile is strictly speaking not the same thing as concrete and goal-oriented behavior such as searching for a job and it is therefore not self-evident that they should really be both captured by the same concept.

More importantly, the fact that the attitude criterion captures both symbolic acts and concrete behavior also creates an overlap—or an insufficient differentiation—with the reciprocity criterion. This criterion is defined as the extent to which a person or group has made contributions to others in the past (e.g., by having worked and paid taxes) or to which she currently engages in efforts to contribute—such as engaging in active job-search (van Oorschot & Roosma, 2017, p. 14; compare also the definitions used by Petersen, 2012; Petersen et al., 2010). As a result, behavior such as active job-search would currently count towards fulfilling both the attitude and the reciprocity criterion simultaneously, creating an overlap between the two criteria (Reeskens & van der Meer, 2019, p. 172 find also that the two criteria are “intertwined”).

The currently broad definition of the reciprocity criterion as referring to both past and current behavior can also be questioned. Obviously, and as in the case of the attitude criterion, the current definition is not illogical. However, recent qualitative research has found that people do in fact hold past and current reciprocal behavior apart and consider them separately when determining others’ deservingness (Heuer & Zimmermann, 2020; Laenen et al., 2019). The current broad definition of the reciprocity criterion does not account for this.

These conceptual issues also lead to real inefficiencies in applied research, specifically varying operationalizations and thus lacking comparability of results across studies. To demonstrate this, we have conducted a review of the recent empirical research on deservingness perceptions with a focus on how the criteria for perceived deservingness are operationalized. Our review included well-known publications such as the early articles by van Oorschot (2000, 2006, 2008) and the recent volume by van Oorschot et al. (2017), but we also conducted an online search for related publications by other authors using a set of search terms.^1^ Additionally, we included publications that were cited in the studies we reviewed and seemed relevant. The corpus of publications on which our review is based is presented in the online supplement.

A first notable finding is that there is indeed often an overlap between the operationalizations of the attitude and reciprocity criteria, which reflects the overlap between their definitions. Reeskens and van der Meer (2017, 2019), for example, operationalize the attitude and reciprocity criteria jointly as the degree to which a fictional unemployment benefit claimant engages in both job-search activities and volunteering.

We further find considerable variation in how the reciprocity criterion itself is measured across different studies. In his first study on deservingness perceptions, van Oorschot (2000) uses age (being a pensioner vs. being young) and the duration of past employment to measure the reciprocity criterion, while for instance Kootstra (2016) uses only the latter. Relatedly, other studies have operationalized the degree to which persons reciprocate variably as their current activities (e.g., active job search) or past behavior such as whether they have previously been economically active (Aarøe & Petersen, 2014; Petersen, 2012; Petersen et al., 2010).

Moving beyond the two criteria of attitude and reciprocity, we also find considerable variation and overlaps when it comes to the operationalizations of the other criteria for perceived deservingness. The control criterion, for instance, is in some studies operationalized as the reason for a benefit claimant’s current situation (e.g., whether or not unemployment was self-induced, or whether lifestyle choices contributed to poor health outcomes), but there are also cases where the control criterion is measured quite differently. Kootstra (2016) for example measures the control criterion via claimants’ efforts to find new employment. A related example is the study by Buss (2019), where age is used to measure the two criteria of control and reciprocity simultaneously. Finally, van der Aa et al., (2017, p. 245), in their study of deservingness perceptions in the case of health care, use two separate indicators (patients’ behavior before falling ill and their behavior during treatment) to simultaneously measure the attitude and control criteria.

We want to stress that our aim here is not to question the quality of these studies or the validity of their findings. Instead, we want to point out that the partly ambiguous definitions of the criteria for perceived deservingness produce problematic outcomes: First, the fact that the operationalizations of the various criteria differ so strongly between studies makes it obviously difficult to directly compare these studies’ findings. Second, the fact that operationalizations overlap even within studies also makes it difficult to interpret the findings of any single study. For instance, if one were to measure the criteria for attitude and reciprocity with the same single indicator, such as engagement in active job search, it would not be clear if any significant effect of this indicator should be interpreted as confirming the relevance of the attitude or the reciprocity criterion (or both).

Accordingly, deservingness research could benefit from more clear-cut definitions of the different deservingness criteria and thus greater clarity about how they should be operationalized in a consistent way within and across studies.

## A Reformulation of Deservingness Criteria

In the following, we develop a redefined set of deservingness criteria that reduces the overlaps between and the heterogeneity within the different criteria, focussing especially on the criteria of attitude and reciprocity.

As above, we start with the attitude criterion and address its internal inconsistency and overlap with the reciprocity criterion. Our suggestion to resolve these issues is straightforward: We suggest defining the attitude criterion narrowly (and arguably more in line with its original meaning) as capturing only symbolic expressions and gestures that signal gratitude, for example a smile. By implication, the attitude criterion should not refer to any behavior that is aimed at more concrete outcomes (e.g., searching for a job or volunteering). To put this more bluntly, the attitude criterion should refer exclusively to “talk”, not to “action”.

Behavior other than expressions of gratitude should accordingly be captured by other criteria. A first relevant category of behavior to be allocated is behavior that in some way involves making reciprocal contributions to others. In the CARIN framework as it is currently used, such behavior (both past and present) would be captured by the reciprocity criterion. Based on the discussion above, however, we suggest that a differentiation should be made between reciprocal behavior that occurred in the past and such behavior if it occurs in the present by using separate criteria. A first criterion would capture past reciprocal acts, for instance past employment, tax payments, or volunteering activities. To stick the established terminology, we suggest using the label “reciprocity” for this criterion.

A new and separate criterion should capture activities or behaviors that are currently being performed and that may aim at making contributions in the future. Examples include current volunteering, actively seeking work when unemployed or complying with treatment protocols when sick. To reflect that these activities signal that effort is being made to either directly contribute to others or to enable oneself to contribute in the future (e.g., by finding work and then paying taxes), we label this criterion “effort”. Separating past and current reciprocal behavior in this way resolves the ambiguities outlined above and, more importantly, reflects the results from recent qualitative research on the formation of deservingness perceptions (Heuer & Zimmermann, 2020; Laenen et al., 2019).

A third type of behavior includes (non-)actions that contributed to a situation of need and hardship. Examples of such behavior include voluntarily resigning from one’s previous job or adopting or failing to change habits that increase the risk of getting ill. In line with previous research (Petersen et al., 2010; van Oorschot, 2000), we capture such behavior with the “control” criterion. Nevertheless, we stress that the control criterion should only capture behaviors (or the absence thereof) that have caused need and hardship; behaviors that intend to contribute to others in some way would instead fall under the criteria of effort or reciprocity.

In the cases of the next two criteria (need and identity) we also adopt the established definitions. The criterion of need is defined it as the degree of hardship experienced by the person asking for aid.^2^ Aspects that are relevant here include for instance the current gap between one’s costs of living and the available resources, or also the number of dependents one is responsible for.

We define the identity criterion as the degree to which a person requesting aid is similar in her social identity to the person evaluating her deservingness (van Oorschot, 2000). Importantly, to avoid any overlaps with the other criteria, we suggest that the identity criterion should not refer to any behavior, expression of an attitude or level of need. At the same time, it is also important to be attentive to the fact that its precise meaning may vary from situation to situation. In some cases, immigration background, skin color, or ethnicity may designate group boundaries (Alesina et al., 2018; Eger, 2010) whereas social class or gender may be more relevant in other situations (e.g., Achterberg et al., 2014).

Table 1 summarizes our proposed set of criteria, compares it to the CARIN criteria and thereby highlights the main changes we introduce. The first change is a clear separation between the criteria of attitude and reciprocity, and the second is a differentiation between reciprocal behavior that occurred in the past and current behavior. In the cases of the other three criteria, our contribution is admittedly more limited and consists of a restatement and clarification.

**Table 1:** Conceptualizations of deservingness criteria

Previous conceptualization		Revised conceptualization	
Criterion	Definition	Criterion	Definition
Control	Situation of hardship caused by one’s own (in)action?	Control	Situation of hardship caused by one’s own (in)action?
Attitude	Gratitude and docility as symbolic *or goal-oriented* acts	Attitude	Gratitude and docility as symbolic gestures
Reciprocity	Acts that contribute to others in past and present	Reciprocity	Past acts that contribute to others
		Effort	Current acts that contribute to others in the future
Identity	Degree of shared membership in social groups	Identity	Degree of shared membership in social groups
Need	Degree of hardship experienced	Need	Degree of hardship experienced

Our conceptualization provides two major benefits. First, the narrower definitions remove ambiguity when it comes to choosing operationalizations in empirical analyses. Second and relatedly, the results of empirical analyses also become more straightforward to interpret. This, in turn, leads to clearer conclusions about which criteria really drive deservingness perceptions and which are more important than others.

## Experiments

To demonstrate how the application of our conceptualization leads to more clear-cut conclusions about which criteria drive deservingness perceptions, we conducted three survey experiments in the US and Germany in 2019.

Specifically, we conducted vignette rating experiments, which are a type of multidimensional choice experiment, similar to conjoint designs (e.g., Hainmueller et al., 2015). This type of experiment is nowadays commonly used in deservingness research and other fields (e.g., Ford, 2016; Kootstra, 2016; Reeskens & van der Meer, 2019) because it produces unbiased estimates of the effects different criteria on deservingness perceptions.

In our vignette survey experiments, respondents were presented with a sequence of eight brief descriptions (“vignettes”) of fictional unemployment benefit claimants. Each vignette contained information about the extent to which a given claimant fulfilled the different deservingness criteria. Crucially, the information relating to the deservingness criteria presented on each individual vignette was varied at random.^3^ This random composition ensured that the unique and unbiased causal effect of each criterion on deservingness perceptions could be estimated. In addition, vignettes were randomly assigned to participants, which ensured that our effect estimates were not influenced by unobserved participant attributes (see also Atzmüller & Steiner, 2010, or Jasso, 2006 for further details on the methodology).

Respondents were asked to indicate how deserving each individual claimant was by stating what percentage of their former salary they should receive. Answers could range from 0 to 100 on a sliding scale. These evaluations were used as the dependent variable in our analyses.

All fictional claimants were introduced as being male, 35 years old, and having earned an average income before becoming unemployed. By holding these demographic attributes constant, we focus the analysis on the core deservingness criteria, which were operationalized as follows (see also Table 2). The control criterion was operationalized as the reason for why a given claimant became unemployed(whether they resigned voluntarily or were dismissed by their employer), the reciprocity criterion was operationalized as the extent to which claimants paid social security contributions in the past, and the effort criterion was measured as how intensely claimants are currently looking for work. The latter two operationalizations reflect our new definitions of the reciprocity and effort criteria in that we used past behavior to operationalize the reciprocity criterion and current behavior to operationalize the effort criterion.

**Table 2:** Vignette attributes

Criteria	Levels	Description
Control		“…has become unemployed because…”
	1	“…his company had to lay off workers”
	2	“…he resigned voluntarily”
Attitude		“…sees unemployment benefits…”
	1	“…as an entitlement he has earned by paying taxes”
	2	“…as generous aid he is thankful for”
Reciprocity		“Before becoming unemployed, he paid social security contributions for X years.”
	1	“one”
	2	“two”
	3	“four”
	4	“eight”
Effort	1	“is not looking for a job currently”
	2	“is looking for a job and is sending out 1–2 applications per week”
	3	“is looking for a job and is sending out 3–4 applications per week”
	4	“is looking for a job and is sending out 5–6 applications per week”
Identity		“Was born in…”
	1	“the United States”/“Germany”
	2	“Canada”/“Austria”
	3	“Mexico”/“Italy”
	4	“Vietnam”/“Romania”
	5	“Pakistan”/“Morocco”
Need	1	“Is financially responsible only for himself”
	2	“Is financially responsible for his partner”
	3	“Is financially responsible for his partner and their common child”
	4	“Is financially responsible for his partner and three children”

We also devised an operationalization of the attitude criterion that corresponds to our definition, i.e., one that captures solely the fictional benefit claimant’s attitude and not any other behavior or attribute. Our approach was to provide information about the claimant’s perception of how entitled they are to receive benefits. In one case, the claimant was presented as perceiving unemployment benefits as a generous aid that they want to rely on as little as possible. In the other case, the claimant was presented as thinking that they are entitled to receive benefits because they paid taxes and contributions before becoming unemployed. Both claimant types clearly vary in the degree to which they express gratefulness and docility. Thus, if the attitude criterion did matter, we should find a significant difference in perceived deservingness between the two claimant types.

To operationalize the identity criterion, we used the claimant’s country of birth. This operationalization obviously captures only one of several possible ways to have a distant social identity, but immigrant background is an aspect that has had continuously high political and social salience in recent years (e.g., Alesina et al., 2018; Reeskens & van der Meer, 2019). In selecting countries of origin, we chose nationalities that are well represented in the two countries’ populations and increasingly culturally distant from the native population. For the experiments conducted in the US, we selected as possible countries of birth the US, Canada, Mexico, Vietnam and Pakistan. For the experiment conducted in Germany, we chose Germany, Austria, Italy, Romania, and Morocco. Finally, we operationalized the need criterion as the number of dependents the fictional claimant is financially responsible for. This operationalization corresponds to those used in many previous studies on deservingness perceptions (e.g., Kootstra, 2016; Reeskens & van der Meer, 2019).

We employed this design in three separate experiments. For the first experiment, which served only as a pre-test, we relied on a sample of US-based respondents (*N* = 334) who we recruited via the Amazon Mechanical Turk (AMT) online platform.^4^ The sample we obtained was disproportionately young, male, and college-educated compared to the general US population. We report the findings from this experiment only briefly here in the main text and present the detailed results, along with descriptive statistics for our samples, in our online supplement.

Following this pre-test, we administered our experiment two more times on new respondent samples, which we recruited from the online respondent pools operated by Qualtrics in the US (*N* = *360*) and Germany (*N* = *400*).^5^ Both samples were selected to resemble the general populations in the US and Germany in terms of the distribution of age groups, genders, and educational attainment via quotas (we provide sample demographics and comparisons to official census data in our supplement). All our experiments were conducted in October and November of 2019; our results are thus not influenced by the COVID-19 pandemic.

By conducting our experiments in the US and Germany, we accounted for the possibility that some macro-level factors could plausibly produce differences in the extent to which different criteria drive deservingness perceptions. For one, both countries have developed different welfare state institutions (Esping-Andersen, 1990) and economic models (Hall & Soskice, 2001): Germany is typically seen as an archetypical case of a conservative welfare state and coordinated market economy, in which the government intervenes relatively strongly into markets and where there is a strong and extensive social safety net (albeit one with a pronounced tendency to maintain existing class and gender inequalities). The US, in contrast, are seen as a clear case of a liberal welfare state and economic model, where markets generally enjoy primacy over the state, including in the area of social protection. In addition, the US are also traditionally more racially and ethnically diverse than Germany. Others have previously argued that all these factors influence not only attitudes toward the welfare state in general but also perceptions of benefit claimants and the relevance of different criteria for deservingness specifically (Alesina & Glaeser, 2004; Gilens, 1996; Larsen, 2008b).

In our statistical analysis, we regressed respondents’ deservingness ratings on the entire set of vignette attributes, which were entered into the model as dummy variables. To account for the hierarchical nature of our data (each respondent rated eight separate vignettes) in our estimations, we used linear random-effects regression models, which we fitted separately on each sample. Our models also included several respondent-level controls, specifically gender (female versus male), age, highest level of education attained, income, and (in the US) race or ethnic background. We present the coefficient estimates for our vignette attributes—i.e., the different deservingness criteria—and 95% confidence intervals in graphical form here. The detailed results, including the estimated effects of the respondent-level variables, are presented in our online supplement.

## Results

**Fig. 1:**
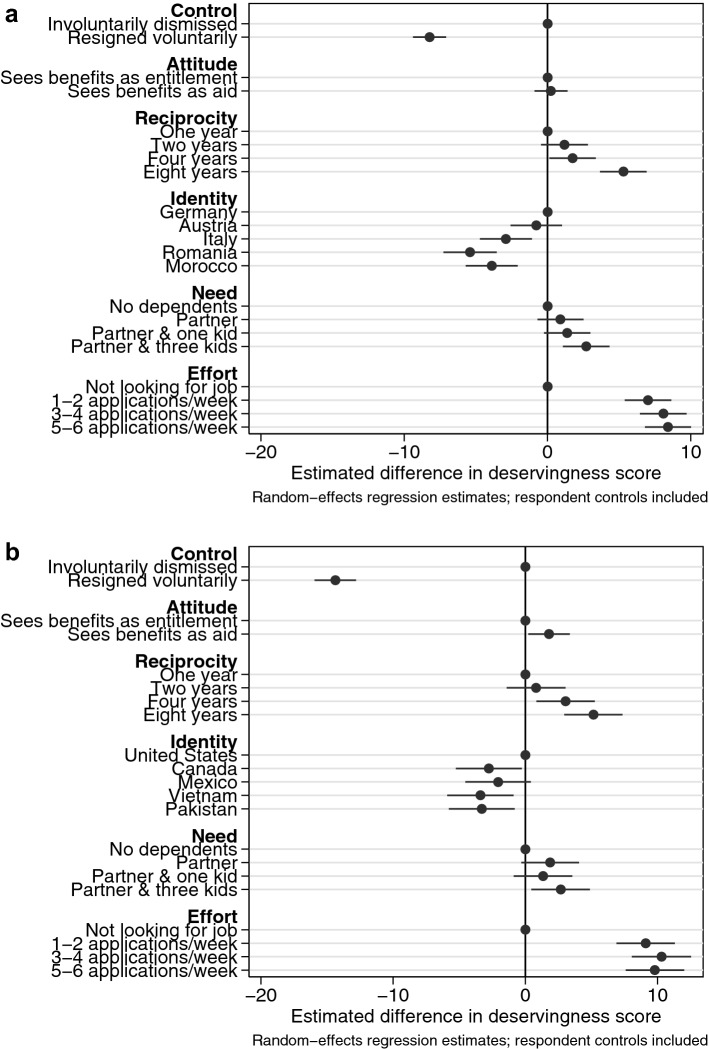
The estimated effects of deservingness criteria

Figure 1 displays the estimated effects of the different criteria on deservingness perceptions in the US (panel b) and Germany (panel a). The control criterion and our new effort criterion had the strongest effects on respondents' evaluations: claimants who resigned voluntarily from their previous jobs or who make no effort to find new work were seen as about ten points less deserving, i.e., should receive a benefit that is ten percentage points lower. These effects were clearly statistically significant, and similar across the two samples. The reciprocity criterion also had strong effects, although the patterns differed somewhat between the US and Germany. An unemployment benefit claimant who has paid taxes and contributions for two years was not seen as significantly more deserving in either country than a claimant who has paid taxes for only one year. A contribution record of four years, however, produced a significantly raised deservingness perception in the US but not in Germany. Finally, claimants who have paid taxes for eight years were seen as significantly more deserving than those who have paid taxes for only one year in both countries. We add that these patterns were similar in our pre-test based on the AMT sample.

Benefit claimants with an immigrant background were seen as less deserving than natives, although there was again some variation between the different countries of origin and between the two samples. In Germany, Austrians were not seen as significantly less deserving than Germans, but all other immigrant groups were. In the US, it was Mexicans who were on par with US natives while immigrants from all other countries (including Canadians) were seen as less deserving. In our initial experiment based on our AMT sample, a foreign background had a consistently negative but not statistically significant effect. This might have been a result of the fact that the sample included many young and college-educated respondents.

The need criterion had ambiguous effects across the different samples. In both Germany and the US, having a dependent partner or a partner plus a child did not significantly raise one’s perceived deservingness to benefits. Only the most extreme case, a claimant with a dependent partner and three children, was rated as significantly more deserving. However, these effects  changed somewhat across our estimations depending on whether or not we included respondent-level controls (see the online supplement). In our pre-test, we furthermore found a significant effect for couples with one child, adding to the overall ambiguous picture.

Moving finally to the attitude criterion, whether claimants display gratefulness had a very limited effect on deservingness perceptions. The estimated effect of the attitude criterion was visibly smaller than the effects of the other criteria, and it was statistically insignificant in the German sample (and the pre-test). In the US, it was only barely statistically significant.

So far, these results already provide a clearer picture of which deservingness criteria really matter. Notably, they show that when the attitude criterion is operationalized strictly as an expression of gratefulness and docility and separate from any behavior, it has no real effect on deservingness perceptions. In contrast, the criteria of effort and reciprocity both had strong and significant effects, which suggests that people really do consider both types of reciprocal behavior separately when making deservingness evaluations, and which is in line with findings from previous qualitative studies (see above). Overall, the weak performance of the attitude criterion and the strong effects of the criteria of reciprocity and effort indicate that people tie deservingness perceptions to “action”, not to “talk”.

In an additional step, we also assessed the importance of the different criteria more generally. In essence, we tested more explicitly which criteria an empirical model of deservingness perceptions really needs to include and which can be left out. To do so, we compared the performances of a set of different model specifications that included varying combinations of deservingness criteria. The baseline was a model containing the core CARIN criteria, i.e., control, attitude, reciprocity (as we have defined it), identity and need. We also considered more parsimonious models that resemble the specifications used in some earlier studies, where only the effects of the control, reciprocity or effort criteria were considered (Aarøe & Petersen, 2014; Petersen, 2012; Petersen et al., 2010, 2012).^6^ Our primary alternative specification was a model that was based on our previous results, and which included the criteria of need, identity, control, effort and reciprocity (or NICER).

We first compared the performances of different nested specifications using likelihood-ratio tests. The results are presented in Table 3 (those based on the data from our pre-test are again presented in the online supplement). The first comparisons were between very restricted models that included only the criteria of control and reciprocity (C + R) or control and effort (C + E) and the NICER specification. The latter specification was clearly preferred in all cases. Second, we compared the NICER specification to one that included also the attitude criterion, i.e., a CARINE specification. This specification was only preferred to the NICER specification in the main US sample but not in the German sample (nor in the pre-test sample). Note also that the chi-squared statistic of this test was still relatively low in the US sample. Overall, these results suggest again that adding the attitude criterion does not do much to improve model performance.

**Table 3:** Model comparisons based on likelihood-ratio tests

Comparison	*p* value	Degrees of freedom	Chi-squared
(a) German sample			
C + E versus NICER	0.000	10	114.22
C + R versus NICER	0.000	10	185.80
NICER versus CARINE	0.295	1	1.10
(b) US sample			
C + E versus NICER	0.000	10	52.53
C + R versus NICER	0.000	7	138.48
NICER versus CARINE	0.020	1	5.42

Finally, we conducted a direct test of a specification including the core CARIN criteria against the NICER specification. These two specifications are non-nested, and we therefore used model information criteria instead of likelihood-ratio tests. Specifically, we relied on the Bayesian and Akaike’s information criteria (BIC and AIC), where smaller scores indicate a better model performance. The results are shown in Table 4. In both samples, the NICER specification produced slightly but consistently better test scores (this was also the case when we ran the test on the pre-test sample). We take this as a further indication that the NICER specification produces a better fit to the data, arguably due to the weak performance of the attitude criterion.

**Table 4:** Direct model comparisons using information criteria

	CARIN	NICER
(a) German sample		
*N*	3168	3168
AIC	27,267.2	27,139.6
BIC	27,358.1	27,242.7
(b) US sample		
*N*	2848	2848
AIC	25,395.4	25,280.2
BIC	25,484.7	25,381.4

## Conclusion

We have addressed a key problem in current research on deservingness perceptions, namely ambiguously defined criteria for perceived deservingness. To resolve this issue, we have suggested a more straightforward conceptualization of these criteria and illustrated how it can be applied empirically.

We have also provided a clearer picture of which deservingness criteria really matter and which do not. Our results indicate that the attitude criterion, if defined and operationalized strictly as expressions of gratitude and docility and separate from any reciprocal behavior, has no consistent effect on deservingness perceptions. In other words, it is likely that previous analyses that did find an effect of the attitude criterion but relied on operationalizations that overlapped with the reciprocity criterion (e.g., Reeskens & van der Meer, 2017) have really captured the effect of the reciprocity criterion. Our results also suggest that it makes sense to have separate criteria for different types of reciprocal behavior: One for actions that occurred in the past, and one for current behavior. As mentioned, this is in line with the findings from recent qualitative research (Heuer & Zimmermann, 2020; Laenen et al., 2019).

This being said, we also want to point out some limitations of our study. First, our analysis covered only two of the three major welfare regimes—the liberal and conservative ones—and leaves out the Northern European or social democratic regime. We can thus not say with the same level of certainty whether our findings would also travel to, say, Sweden or Denmark. Nevertheless, there are reasons to believe that deservingness perceptions do not differ that much between welfare regimes. Our own analysis revealed quite similar patterns in the US and Germany, and these findings resemble those of Aarøe and Petersen (2014), who showed that welfare deservingness perceptions are formed in highly similar ways by both Americans and Danes. In addition, many important patterns in deservingness research are generally similar across countries. One such pattern, for instance, is that immigrants are seen as less deserving per se (e.g., Alesina et al., 2018; Reeskens & van der Meer, 2019). It is therefore likely that our findings can also be applied to contexts other than those we have studied here. Additional research is obviously needed to verify this.

A second limitation is that we could assess the relevance of our new set of deservingness criteria only in relation to deservingness to unemployment benefits and not to other types of cash benefits or services. Some previous studies have indeed shown that deservingness perceptions may be formed differently in the case of health care than in the case of unemployment benefits. Van der Aa et al. (2017), for instance, show that (medical) need strongly affects deservingness to health care, while as we have shown here, the need criterion is less important in the case of unemployment benefits. The findings by Jensen and Petersen (2017) also suggest that deservingness perceptions are formed in different ways when it comes to the sick compared to the unemployed. In essence, they show that the sick are by default seen as less in control and therefore more deserving than the unemployed.

Nevertheless, even the study by van der Aa et al. (2017) showed that sick patients are penalized if they contributed to their falling ill, thus suggesting that expectations of reciprocal behavior are also at work in the case of health care policies. Similar findings come also from medical research on public attitudes toward the allocation of organ transplants, where patients whose lifestyle choices have contributed to their need for a donor organ are seen as significantly less deserving of receiving one (Ubel et al., 2001). In addition, there is also research showing that people discriminate against those with a foreign social identity even when it comes to medical care (O’Dell et al., 2019). Thus, while there may be some differences concerning the relative importance of different deservingness criteria as regards different areas of the welfare state, we still think that the main dynamics remain the same: when forming deservingness perceptions, people pay attention to levels of need; past acts of reciprocity and current efforts; the extent to which any hardship is out of control; and, for better or worse, perceived social and cultural distance or similarity.

## Supplementary Information

Below is the link to the electronic supplementary material.

Supplementary file1 (PDF 751 kb)

## Data Availability

Our replication data and code files are publicly available at https://osf.io/37me5/.

## Footnotes

1 We used Google Scholar to search for articles using the phrase “deservingness AND CARIN”. We included only articles published in peer-reviewed scholarly journals, and books and book chapters published by academic publishing houses. We excluded all works that clearly had no connection to deservingness perceptions or welfare state research. Not all the studies we reviewed can be clearly categorized as applying the CARIN framework, and many also use other approaches, at least to some extent.

2 Building on Delton et al. (2018), the need criterion could also be divided further into two separate concepts: individuals’ absolute level of need (the amount of resources they need to have available but do not) and individuals’ relative level of need (the difference between their current and previous situation—how “far they have fallen”).

3 Specifically, we drew a D-efficient sample from the entire universe of all possible vignette versions. Since our vignettes featured six criteria of which two had two levels, three had four levels and one had five levels, the overall universe of all possible combinations of vignette attributes included (2 × 2) × (4 × 4 × 4) × 5 = 1280 vignette versions. To minimize the loss of information that goes along with using only a subset of all possible vignettes, we drew a D-efficient rather than purely random sample using the SAS algorithms developed by Kuhfeld (1997, 2010). Our sample orthogonalized all two-way interactions. Our preferred design included 160 vignettes overall, which were divided into 20 decks of 8 vignettes each to ensure that all vignettes are rated by a sufficiently large number of participants. This design achieved a D-efficiency of 90.0335 and therefore met the rule-of-thumb threshold for design D-efficiency values of above 90 (see also Auspurg & Hinz, 2015, pp. 38–40). We did not identify any illogical combinations of vignette attributes.

4 See also our supplementary materials for details on how respondents were recruited and remunerated. From our initial AMT sample, we dropped 15 respondents who had an overall completion rate of less than 99% and a further six whose responses we judged to be of too low quality (implausible combinations or short response times and little variation in responses). In the end, we retained 313 respondents (corresponding to 2504 vignette ratings) for our analysis.

5 We again dropped some observations that, in our view, had implausibly high incomes (e.g., more than 80.000 EUR per month was reported by a German respondent with basic education) or showed certain response patterns (very short response times in combination with very little variation in vignette ratings) and ended up with 356 and 396 respondents in the US and Germany, respectively, for our analysis.

6 Note that these studies use different experimental setups, which we could only approximate with our model specifications.
