# Supplementary material for: A Recast Framework for Welfare Deservingness Perceptions
Source: Soc Indic Res. 2021 Aug 20;159(3):927–43. doi: 10.1007/s11205-021-02774-9 (PMC8378786; doi:10.1007/s11205-021-02774-9)
Supplement: Supplementary file 1 — Supplementary file1 (PDF 751 kb) [file 11205_2021_2774_MOESM1_ESM.pdf]

SUPPLEMENTARY MATERIALS

Knotz, Carlo M., Mia K. Gandenberger, Flavia Fossati, Giuliano Bonoli. "A Recast Framework for Welfare Deservingness Perceptions" *Social Indicators Research*

1. Recruitment procedure & ethical reimbursement, US AMT sample

When recruiting respondents via AMT, we relied on the *Guidelines for Academic Requesters.*[1] Participants ("workers") were eligible if they had already completed more than 1000 tasks on AMT and had an approval rating of their work of at least 97% to ensure a certain level of response quality. After selecting the task, workers meeting these criteria were presented with a small introductory text and a link to the survey, which was hosted by Qualtrics. The workers were offered 1.40 USD for participating; this payment corresponds to an hourly wage of approximately 12 USD and is a relatively generous remuneration for AMT tasks in the US.

2. Sample characteristics and results, US AMT sample

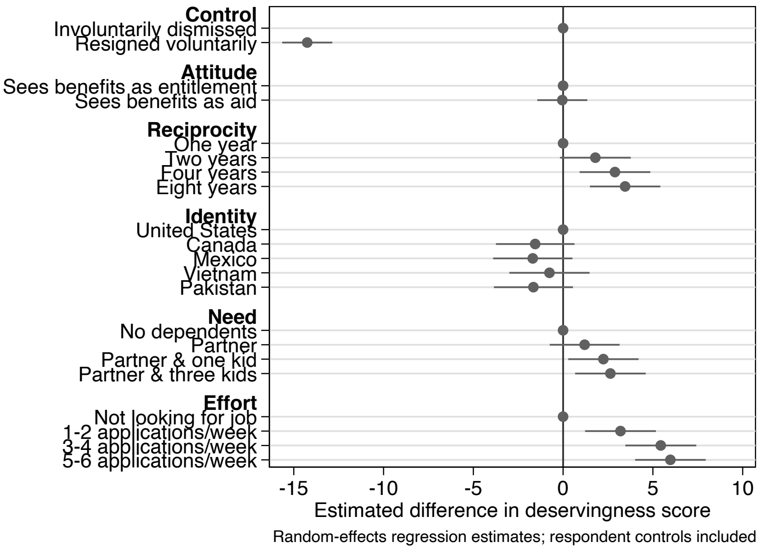

Figure S1: Estimation results based on AMT Sample

[1] Accessible at https://wearedynamo.fandom.com/wiki/Guidelines_for_Academic_Requesters, last accessed on 7 November 2019

	(1)		(2)	
**Vignette variables:**				
Involuntarily dismissed	0.00	(.)	0.00	(.)
Resigned voluntarily	-14.26*	(0.71)	-14.29*	(0.72)
Sees benefits as entitlement	0.00	(.)	0.00	(.)
Sees benefits as aid	-0.05	(0.71)	0.02	(0.71)
One year	0.00	(.)	0.00	(.)
Two years	1.80	(1.00)	1.85	(1.01)
Four years	2.90*	(1.00)	2.95*	(1.01)
Eight years	3.46*	(1.00)	3.52*	(1.01)
United States	0.00	(.)	0.00	(.)
Canada	-1.55	(1.12)	-1.59	(1.12)
Mexico	-1.66	(1.13)	-1.71	(1.13)
Vietnam	-0.74	(1.14)	-0.80	(1.15)
Pakistan	-1.62	(1.12)	-1.67	(1.13)
No dependents	0.00	(.)	0.00	(.)
Partner	1.21	(0.99)	1.16	(1.00)
Partner & one kid	2.24*	(1.00)	2.21*	(1.01)
Partner & three kids	2.64*	(1.00)	2.58*	(1.01)
Not looking for job	0.00	(.)	0.00	(.)
1-2 applications/week	3.18*	(1.01)	3.18*	(1.01)
3-4 applications/week	5.42*	(1.01)	5.52*	(1.01)
5-6 applications/week	5.95*	(1.00)	6.00*	(1.01)
**Respondent variables:**				
Age			-0.05	(0.12)
Female			0.00	(.)
Male			1.69	(2.25)
Less than high school			0.00	(.)
High school or equivalent			-38.86*	(19.09)
Some college or associate's degree			-37.84*	(19.00)
Bachelor's degree			-32.50	(18.92)
Graduate or prof. degree			-26.18	(19.05)
American Indian or Alaska Native			0.00	(.)
Asian			20.82	(19.74)
Black or African American			30.73	(18.98)
Hispanic			18.77	(19.49)
Other			21.84	(20.75)
White			20.29	(19.00)
Constant	58.78*	(1.80)	70.35*	(26.86)
SD(constant)	19.22*	(0.85)	17.63*	(0.80)
SD(residual)	17.62*	(0.27)	17.66*	(0.27)
Observations (vignette evaluations)	2504		2488	
Respondents	313		311	
Chisq. (model p-value)	464.21	(0.000)	511.19	(0.000)

Standard errors in parentheses
* p<.05

Table S1: Detailed estimation results (AMT sample)

Comparison	p-value	Degrees of freedom	Chi-squared
C+E vs. NICER	0.006	10	24.83
C+R vs. NICER	0.000	10	54.38
NICER vs. CARINE	0.942	1	0.01

Table S2: Model comparisons using likelihood ratio tests

	CARIN	NICER
N	2504	2504
AIC	22282.8	22243.8
BIC	22370.2	22342.9

Table S3: Direct model comparison using information criteria

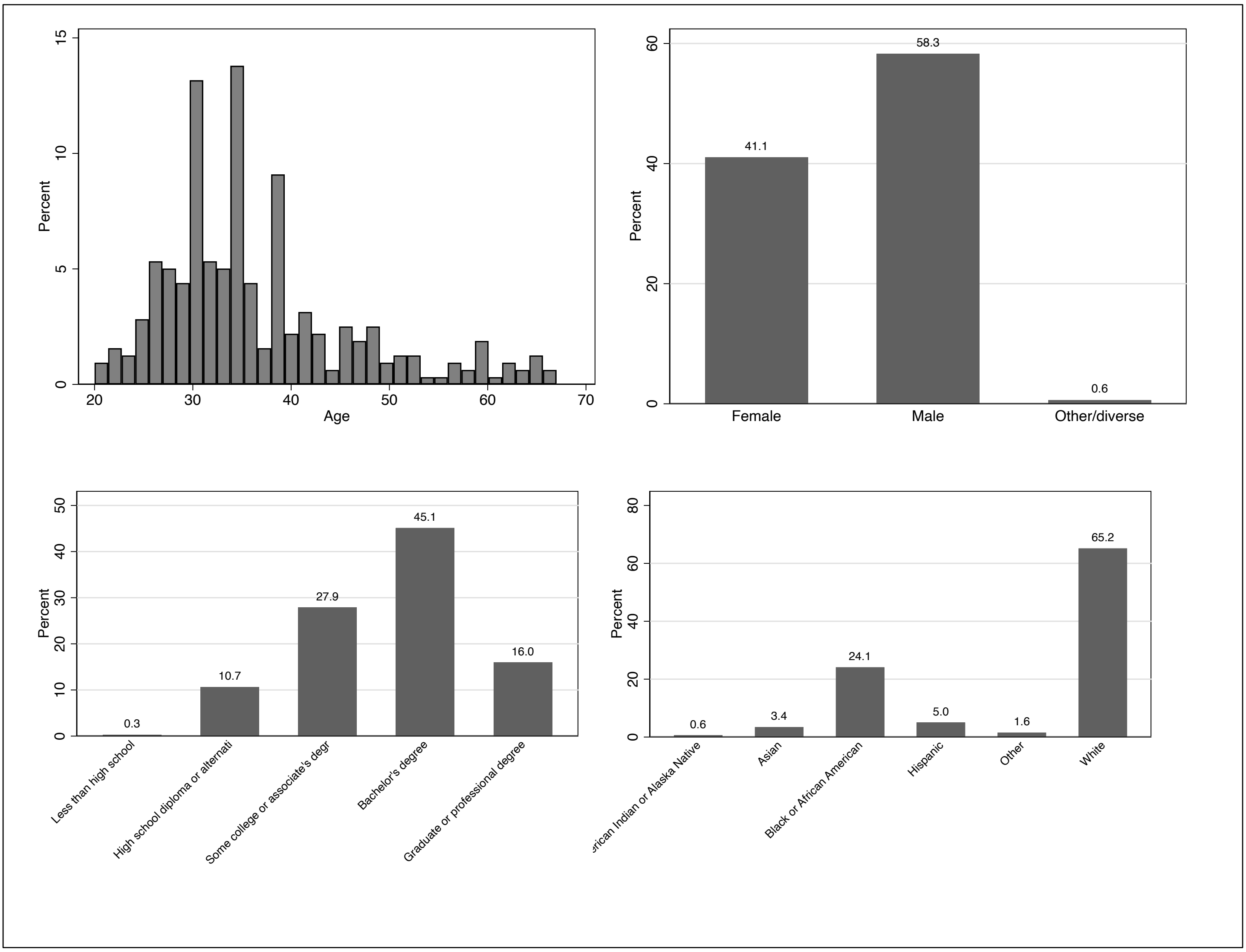

Figure 2: AMT sample characteristics

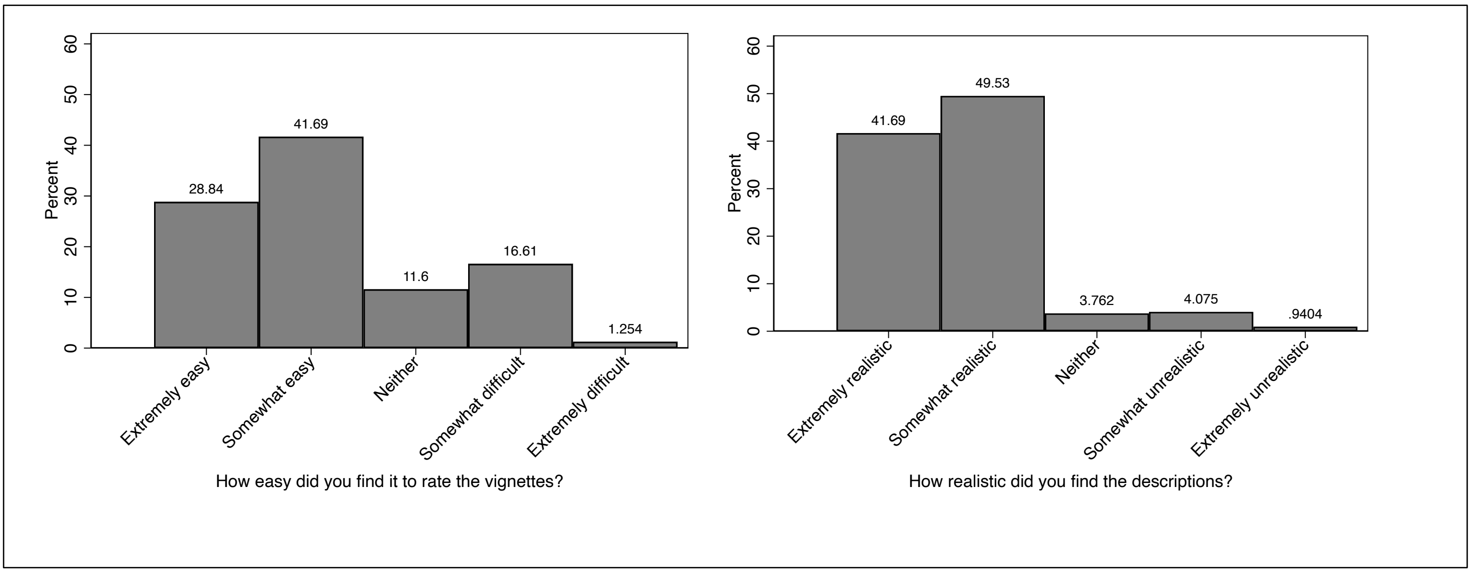

Figure 3: Respondents' evaluations of vignette quality (AMT sample)

3. Sample characteristics & detailed estimation results, main data (US)

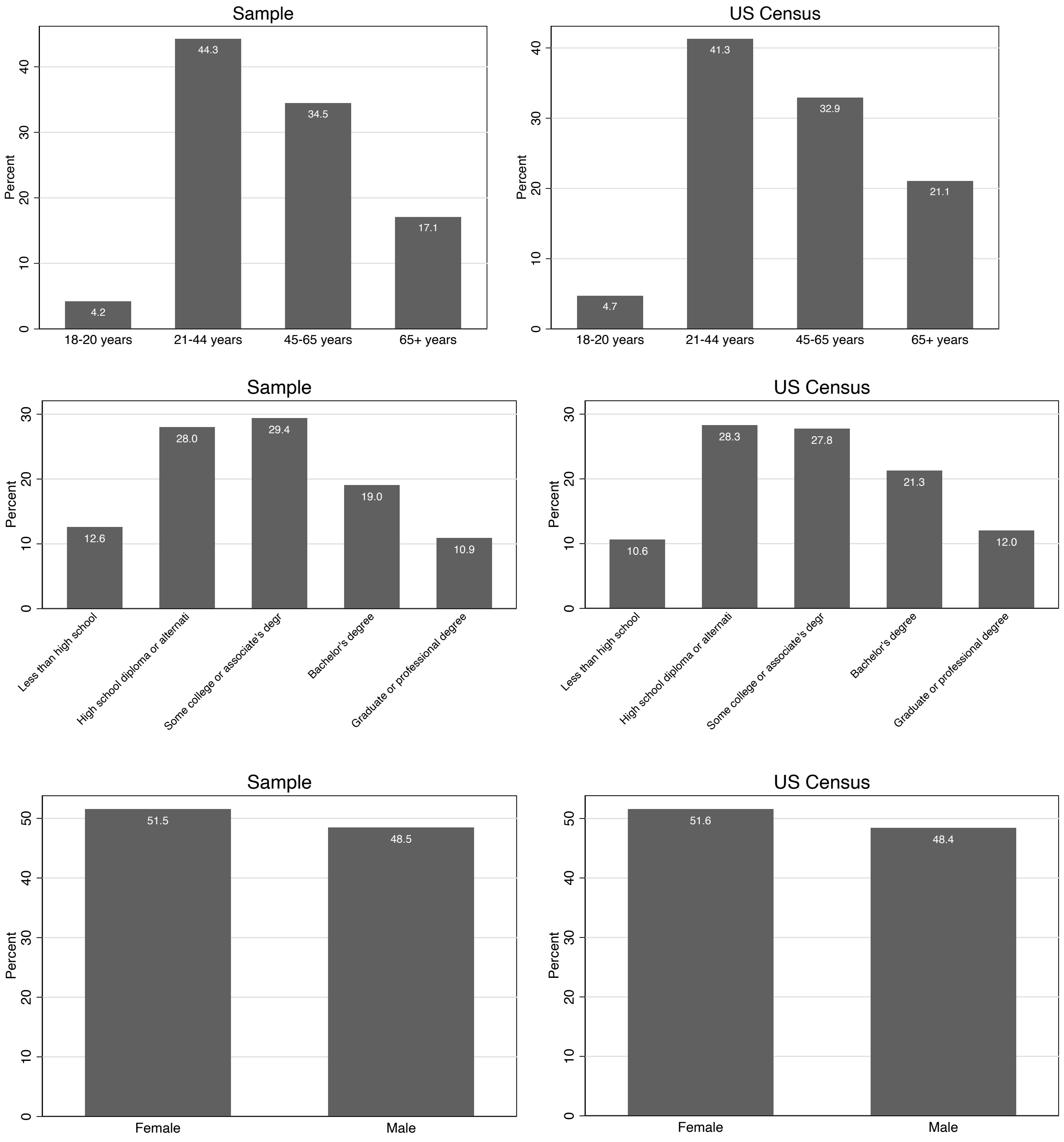

Notes: US Census figures based on 2019 data from https://www.census.gov/data/tables/2019/demo/age-and-sex/2019-age-sex-composition.html; last access on November 27, 2020; all census figures computed for population aged 18 and older. Age groups correspond to age groups used by US Census Bureau.

Figure 4: Sample demographics & US Census comparison

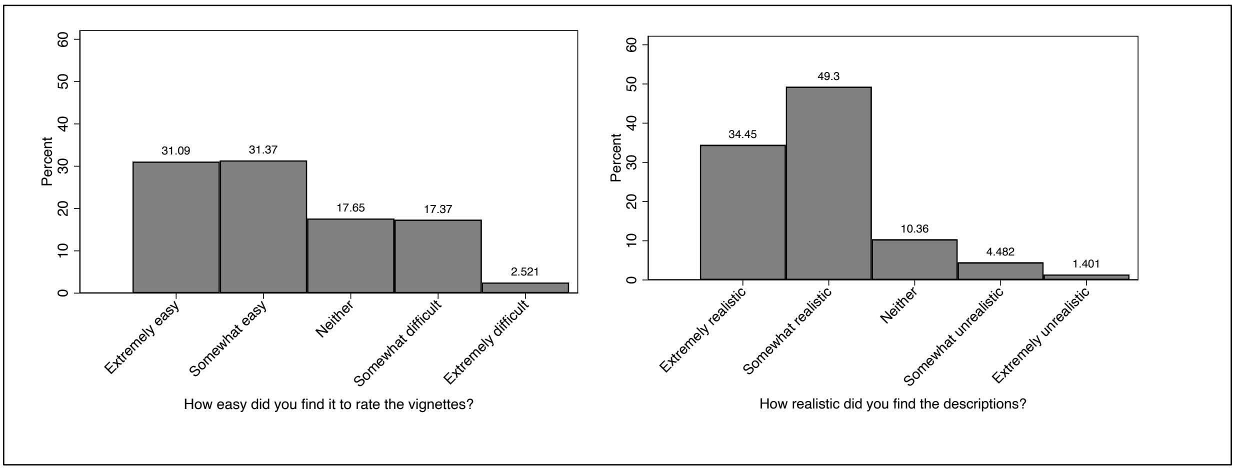

Figure 5: Respondents' evaluations of vignette quality (main data, US)

	(1)		(2)	
**Vignette variables:**				
Involuntarily dismissed	0.00	(.)	0.00	(.)
Resigned voluntarily	-12.45*	(0.65)	-14.37*	(0.80)
Sees benefits as entitlement	0.00	(.)	0.00	(.)
Sees benefits as aid	1.52*	(0.65)	1.80*	(0.80)
One year	0.00	(.)	0.00	(.)
Two years	0.89	(0.92)	0.81	(1.14)
Four years	2.62*	(0.92)	3.05*	(1.13)
Eight years	4.83*	(0.92)	5.15*	(1.12)
United States	0.00	(.)	0.00	(.)
Canada	-2.42*	(1.02)	-2.76*	(1.27)
Mexico	-1.68	(1.03)	-2.06	(1.27)
Vietnam	-3.52*	(1.05)	-3.40*	(1.28)
Pakistan	-3.51*	(1.04)	-3.30*	(1.27)
No dependents	0.00	(.)	0.00	(.)
Partner	1.04	(0.91)	1.88	(1.11)
Partner & one kid	1.70	(0.92)	1.34	(1.13)
Partner & three kids	1.65	(0.92)	2.67*	(1.13)
Not looking for job	0.00	(.)	0.00	(.)
1-2 applications/week	7.66*	(0.92)	9.10*	(1.13)
3-4 applications/week	8.92*	(0.93)	10.30*	(1.14)
5-6 applications/week	8.88*	(0.92)	9.80*	(1.13)
**Respondent variables:**				
Age			-0.19*	(0.10)
Female			0.00	(.)
Male			2.81	(2.95)
Less than high school			0.00	(.)
High school or equivalent			-1.11	(5.01)
Some college or associate's degree			-1.00	(5.14)
Bachelor's degree			-3.22	(5.25)
Graduate or prof. degree			7.21	(6.22)
American Indian or Alaska Native			0.00	(.)
Asian			-16.90	(23.00)
Black or African American			-24.39	(22.34)
Hispanic			-17.80	(22.42)
Other			-24.24	(25.28)
White			-28.42	(21.97)
Income			-0.00	(0.00)
Constant	49.61*	(1.77)	83.58*	(23.01)
SD(constant)	21.90*	(0.88)	20.80*	(1.00)
SD(residual)	17.25*	(0.24)	18.11*	(0.30)
Observations (vignette evaluations)	2848		2096	
Respondents	356		262	
Chisq. (model p-value)	553.54	(0.000)	516.95	(0.000)

Standard errors in parentheses
* p<.05

Table 4: Detailed estimation results (main sample, US)

4. Sample characteristics & detailed estimation results, main data (DE)

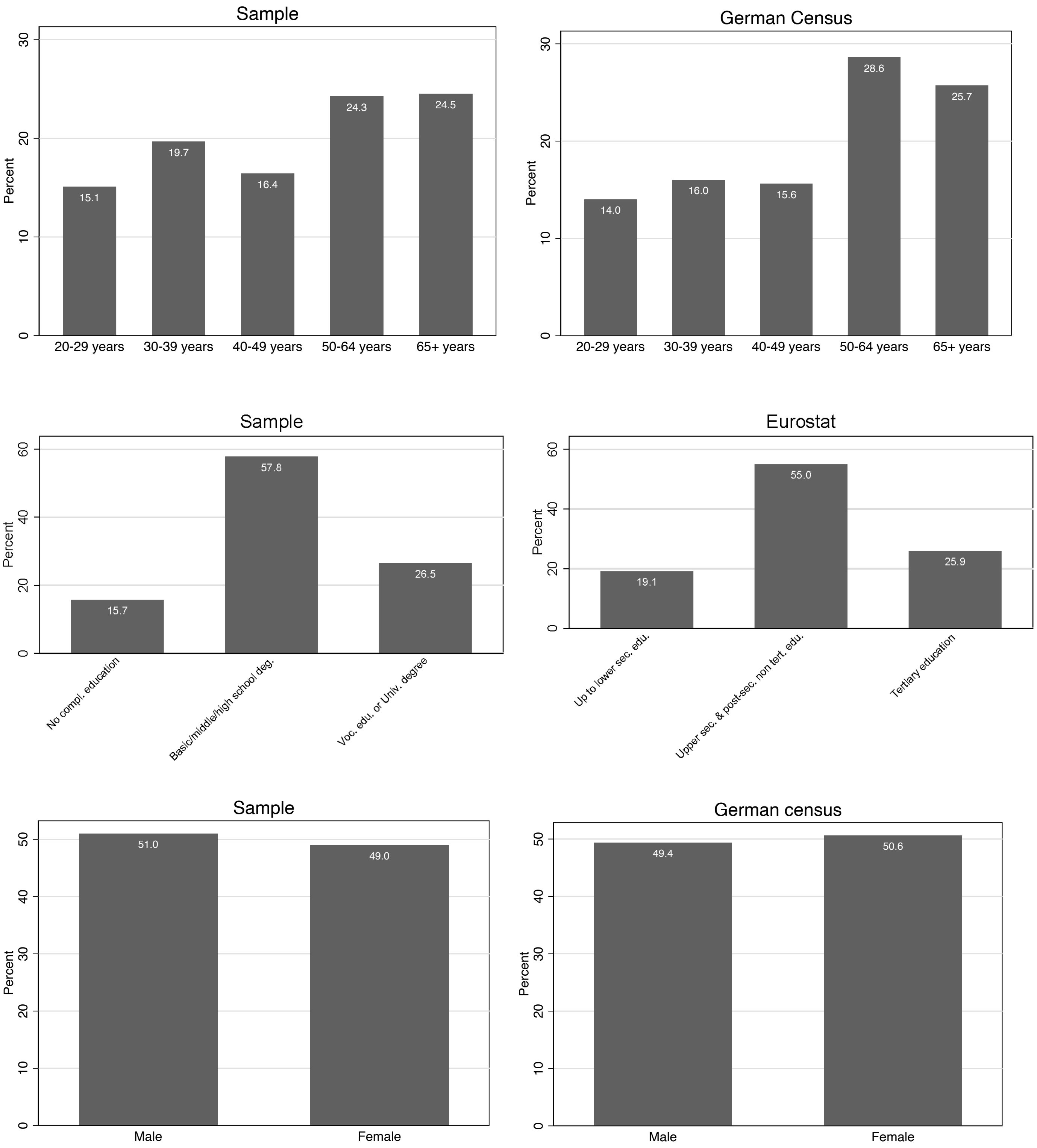

Notes: German census figures based on 2019 data from https://www-genesis.destatis.de/genesis/online?operation=ergebnistabelleUmfang&levelindex=3&levelid=1606481970487&downloadname=12211-0002#abreadcrumb (age) and https://www.destatis.de/DE/Themen/Gesellschaft-Umwelt/Bevoelkerung/Bevoelkerungsstand/Tabellen/liste-zensus-geschlecht-staatsangehoerigkeit.html (gender); both last access on November 27, 2020; Age groups are aggregated to 10-year intervals from census figures, which are in 5-year intervals. The education figures are based on Eurostat data for the year 2019 on the distribution of educational attainment [edat_lfs_9904] for the population between the ages from 15 to 74 (https://ec.europa.eu/eurostat/databrowser/view/EDAT_LFS_9904__custom_306170/default/table?lang=en).

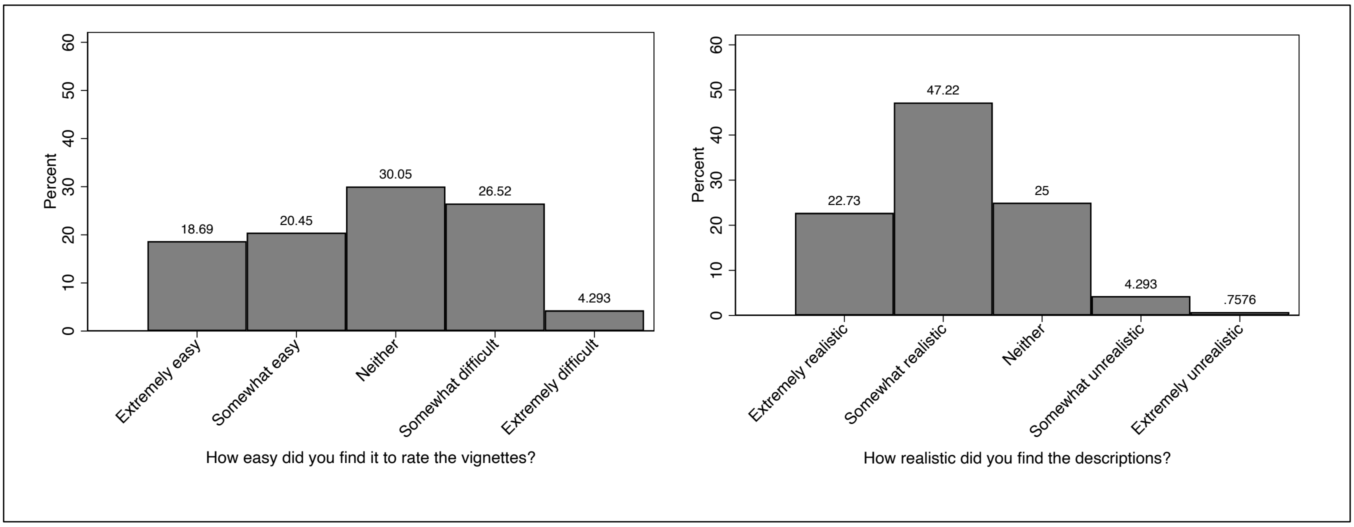

Figure 6: Respondents' evaluations of vignette quality (main data, DE)

	(1)		(2)	
**Vignette variables:**				
Involuntarily dismissed	0.00	(.)	0.00	(.)
Resigned voluntarily	-6.67*	(0.53)	-8.23*	(0.59)
Sees benefits as entitlement	0.00	(.)	0.00	(.)
Sees benefits as aid	0.56	(0.53)	0.24	(0.59)
One year	0.00	(.)	0.00	(.)
Two years	1.89*	(0.75)	1.19	(0.83)
Four years	2.09*	(0.74)	1.75*	(0.82)
Eight years	5.66*	(0.74)	5.29*	(0.83)
Germany	0.00	(.)	0.00	(.)
Austria	-0.16	(0.83)	-0.78	(0.92)
Italy	-1.89*	(0.83)	-2.90*	(0.93)
Romania	-4.61*	(0.85)	-5.40*	(0.95)
Morocco	-4.11*	(0.84)	-3.89*	(0.92)
No dependents	0.00	(.)	0.00	(.)
Partner	0.12	(0.74)	0.91	(0.82)
Partner & one kid	0.99	(0.74)	1.37	(0.83)
Partner & three kids	1.66*	(0.75)	2.70*	(0.83)
Not looking for job	0.00	(.)	0.00	(.)
1-2 applications/week	6.09*	(0.75)	7.02*	(0.83)
3-4 applications/week	7.23*	(0.75)	8.08*	(0.83)
5-6 applications/week	7.60*	(0.74)	8.41*	(0.82)
**Respondent variables:**				
Age			-0.07	(0.07)
Male			0.00	(.)
Female			-0.51	(2.27)
No compl. education			0.00	(.)
Basic or middle school degree			-3.41	(4.56)
Abitur			-0.36	(4.97)
Vocation. education			0.12	(4.93)
University degree			-2.88	(5.23)
Income			-0.00	(0.00)
Constant	55.54*	(1.43)	64.83*	(5.17)
SD(constant)	19.10*	(0.73)	17.52*	(0.81)
SD(residual)	14.77*	(0.20)	13.51*	(0.22)
Observations (vignette evaluations)	3168		2160	
Respondents	396		270	
Chisq. (model p-value)	412.28	(0.000)	425.67	(0.000)

Standard errors in parentheses
* p<.05

Table 5: Detailed estimation results (main data, DE)

			Operationalization			
Study	*Control*	*Attitude*	*Reciprocity*	*Identity*	*Need*	*Notes*
van Oorschot (2000)	Multiple: being unable vs. unwilling to work; disabled as result of work vs. result of own behavior; weak health vs. strong health	Not measured	Pensioners vs. young;<br><br>Work record	Ethnic minority; asylum seeker; illegal aliens	Jobless; single vs. double income household; with vs. without children; low vs. high education	Mentions `social risk' - being sick, widowed, disabled, pensioner - as additional criterion (p. 38)<br><br>Control most important, then Identity, then Reciprocity; Need less important
van Oorschot (2006)	Not directly measured	Not directly measured	Not directly measured	Not directly measured	Not directly measured	Hierarchy of deservingness via comparison between claimant groups (& immigrants)
van Oorschot (2008)	Not directly measured	Not directly measured	Not directly measured	Not directly measured	Not directly measured	Comparison between native claimants and immigrants
Reeskens & van der Meer (2018)	Reason for migration; reason for unemployment	Reintegration strategy (looking for job or not)	Employment record; reintegration strategy	Foreign origin & length of residence	Previous salary, family size	
Kootstra (2016)	Effort to find new job	Not measured	Work history	Ethnic backround, migration status	Having family or not	Does not rely solely on CARIN

Jeene et al. (2014)	Not directly measured	Not directly measured	Not directly measured	Not directly measured	Not directly measured	Hierarchy of deservingness via comparison between claimant groups
Laenen & Meulemann (2017)	Not directly measured	Not directly measured	Not directly measured	Not directly measured	Not directly measured	Hierarchy of deservingness via comparison between claimant groups
Reeskens & van der Meer (2017)	Reason for unemployment (company reorganization vs. unprofessional work attitude)	Volunteering or not	Actively looking for work vs. not looking for work vs. *actively looking for work & volunteering*	Country of origin	Last net salary & family size	
de Vries (2017)	Not directly measured	Not directly measured	Not directly measured	Not directly measured	Not directly measured	Implicit & explicit beliefs about benefit claimants
van Doorn & Bos (2017)	Not directly measured	Not directly measured	Not directly measured	Not directly measured	Not directly measured	News coverage of benefit claimants
Lepianka (2017)	Not directly measured	Not directly measured	Not directly measured	Not directly measured	Not directly measured	News coverage of benefit claimants
Uunk & van Oorschot (2017)	Not directly measured	Not directly measured	Not directly measured	Not directly measured	Not directly measured	Comparison between claimant groups

Buss et al. (2017)	Not directly measured	Not directly measured	Not directly measured	Not directly measured	Not directly measured	Attitudes toward conditionality of unemployment benefits
Roosma & Jeene (2017)	Not directly measured	Not directly measured	Not directly measured	Not directly measured	Not directly measured	Comparison between claimant groups
Blomberg et al. (2017)	Aggrement to: claimants are lazy	Not measured	Aggrement to: claimants have contributed or will contribute to welfare state	Agreement to: anyone can end up needing social assistance	Agreement to: Most of those receiving social assistance really need it	
de Wilde (2017)	Multiple	Multiple	Multiple	Multiple	Multiple	No clear operationalization of criteria; overlaps between criteria
van der Aa et al. (2017)	Lifestyle choices & compliance with treatment	Lifestyle choices & compliance with treatment	Chosen level of health insurance coverage	Not measured	Medical need & financial abilities	
Kootstra (2017)	Agreement to: benefit claimants would not need help if they tried harder	Agreement to: benefit claimants do not appreciate sufficiently that they are receiving taxpayer money	Agreement to: benefit claimants take out more than they contribute	Agreement to: Sympthasize with resp. benefit claimant group	Agreement to: benefit claimants not really in need; spend money reckless	Comparison between perceptions of different enthnic groups
Kumlin et al. (2017)	Not directly measured	Not directly measured	Not directly measured	Not directly measured	Not directly measured	General attitudes toward welfare state, redistribution, welfare chauvinism

Sadin (2017)	Reasons for wealth	Reasons for wealth	Not measured	Not measured	Not measured	
Ragusa (2017)	Stereotypes of rich (e.g. productive & philanthropical vs. greedy & corrupt)	Not directly measured	Stereotypes of rich (hard working & entrepreneurial vs. entitled)	Not directly measured	Not directly measured	Stereotypes of rich; only control & reciprocity really matter
Larsen (2008)	Not directly measured	Not directly measured	Not directly measured	Not directly measured	Not directly measured	Differences in attitudes toward claimant groups (old, working-age, young unemployed)
Laenen et al. (2019)	Inductive measurement	Inductive measurement	Inductive measurement	Inductive measurement	Inductive measurement	Qualitative study; deservingness criteria are attributed to statements from focus group participants
Uunk & van Oorschot (2019)	Not directly measured	Not directly measured	Not directly measured	Not directly measured	Not directly measured	General solidarity with unemployed and its dependence on economic context
Buss (2019)	Age; reason for unemployment	Not measured	Age; being parent	Ethnicity	Being parent	

Table 6: Summary of results of literature review

References

Blomberg H, Kallio J, Kangas O, et al. (2017) Social Assistance Deservingness and Policy Measures: Attitudes of Finnish Politicians, Administrators and Citizens. In: *The Social Legitimacy of Targeted Welfare: Attitudes to Welfare Deservingness*. Cheltenham: Edward Elgar Publishing, pp. 209–224.

Buss C (2019) Public opinion towards targeted labour market policies: A vignette study on the perceived deservingness of the unemployed. *Journal of European Social Policy* 29(2): 228–240.

Buss C, Ebbinghaus B and Naumann E (2017) Making deservingness of the unemployed conditional: changes in public support for the conditionality of unemployment benefits. In: Oorschot W van, Roosma F, Meuleman B, et al. (eds) *The Social Legitimacy of Targeted Welfare: Attitudes to Welfare Deservingness*. Cheltenham & Northampton, MA: Edward Elgar Publishing, pp. 167–185.

de Vries R (2017) Negative Attitudes towards Welfare Claimants: The Importance of Unconscious Bias. In: Van Oorschot W, Roosma F, Meuleman B, et al. (eds) *The Social Legitimacy of Targeted Welfare: Attitudes to Welfare Deservingness*. Cheltenham: Edward Elgar Publishing, pp. 93–110.

De Wilde M (2017) Deservingness in Social Assistance Administrative Practice: A Factorial Survey Approach. In: van Oorschot W, Roosma F, Meuleman B, et al. (eds) *The Social Legitimacy of Targeted Welfare*. Edward Elgar Publishing, pp. 225–240. DOI: 10.4337/9781785367212.00027.

Jeene M, van Oorschot W and Uunk W (2014) The dynamics of welfare opinions in changing economic, institutional and political contexts: an empirical analysis of Dutch deservingness opinions, 1975–2006. *Social Indicators Research* 115(2): 731–749.

Kootstra A (2016) Deserving and Undeserving Welfare Claimants in Britain and the Netherlands: Examining the Role of Ethnicity and Migration Status Using a Vignette Experiment. *European Sociological Review* 32(3): 325–338. DOI: 10.1093/esr/jcw010.

Kootstra A (2017) Us versus Them: Examining the Perceived Deservingness of Minority Groups in the British Welfare State Using a Survey Experiment. In: Van Oorschot, Wim, Roosma F, Meuleman B, et al. (eds) *The Social Legitimacy of Targeted Welfare: Attitudes to Welfare Deservingness*. Cheltenham: Edward Elgar Publishing, pp. 263–280.

Kumlin S, Wollebæk D, Fladmoe A, et al. (2017) Leap of Faith or Judgment of Deservingness? Generalized Trust, Trust in Immigrants and Support for the Welfare State. In: *The Social Legitimacy of Targeted Welfare: Attitudes to Welfare Deservingness*. Cheltenham: Edward Elgar Publishing, pp. 281–296.

Laenen T and Meuleman B (2017) A Universal Rank Order of Deservingness? Geographical, Temporal and Social-Structural Comparisons. In: *The Social Legitimacy of Targeted Welfare*. Edward Elgar Publishing, pp. 37–54. DOI: 10.4337/9781785367212.00012.

Laenen T, Rossetti F and van Oorschot W (2019) Why deservingness theory needs qualitative research: Comparing focus group discussions on social welfare in three welfare regimes. *International Journal of Comparative Sociology* 60(3): 190–216. DOI: 10.1177/0020715219837745.

Larsen CA (2008) The political logic of labour market reforms and popular images of target groups. *Journal of European Social Policy* 18(1): 50–63.

Lepianka D (2017) The Varying Faces of Poverty and Deservingness in Dutch Print Media. In: Van Oorschot, Wim, Roosma F, Meuleman B, et al. (eds) *The Social Legitimacy of Targeted Welfare: Attitudes to Welfare Deservingness*. Cheltenham: Edward Elgar Publishing, pp. 127–145.

Ragusa J (2017) Do the Rich Deserve a Tax Cut? Public Images, Deservingness Criteria and Americans' Tax Policy Preferences. In: Van Oorschot, Wim, Roosma F, Meuleman B, et al. (eds) *The Social Legitimacy of Targeted Welfare: Attitudes to Welfare Deservingness*. Cheltenham: Edward Elgar Publishing, pp. 316–332.

Reeskens T and van der Meer T (2017) The Relative Importance of Welfare Deservingness Criteria. In: van Oorschot W, Roosma F, Meuleman B, et al. (eds) *The Social Legitimacy of Targeted Welfare*. Cheltenham & Northampton, MA: Edward Elgar Publishing, pp. 55–70. DOI: 10.4337/9781785367212.00013.

Reeskens T and van der Meer T (2019) The inevitable deservingness gap: A study into the insurmountable immigrant penalty in perceived welfare deservingness. *Journal of European Social Policy* 29(2): 166–181. DOI: 10.1177/0958928718768335.

Roosma F and Jeene M (2017) The Deservingness Logic Applied to Public Opinions Concerning Work Obligations for Benefit Claimants. In: Van Oorschot, Wim, Roosma F, Meuleman B, et al. (eds) *The Social Legitimacy of Targeted Welfare: Attitudes to Welfare Deservingness*. Cheltenham: Edward Elgar Publishing, pp. 189–205.

Sadin M (2017) They're Not Worthy: The Perceived Deservingness of the Rich and its Connection to Policy Preferences. In: Van Oorschot, Wim, Roosma F, Meuleman B, et al. (eds) *The Social Legitimacy of Targeted Welfare: Attitudes to Welfare Deservingness*. Cheltenham: Edward Elgar Publishing, pp. 299–315.

Uunk W and van Oorschot W (2019) Going with the Flow? The Effect of Economic Fluctuation on People's Solidarity with Unemployed People. *Social Indicators Research* 143(3): 1129–1146. DOI: 10.1007/s11205-018-2023-z.

Uunk W and Van Oorschot, Wim (2017) How Welfare Reforms Influence Public Opinion Regarding Welfare Deservingness: Evidence from Dutch Time-Series Data, 1975–2006. In: Van Oorschot, Wim, Roosma F, Meuleman B, et al. (eds) *The Social Legitimacy of Targeted Welfare: Attitudes to Welfare Deservingness*. Cheltenham: Edward Elgar Publishing, pp. 149–166.

Van Der Aa M, Hiligsmann M, Paulus A, et al. (2017) Healthcare Deservingness Opinions of the General Public and Policymakers Compared: A Discrete Choice Experiment. In: van Oorschot W, Roosma F, Meuleman B, et al. (eds) *The Social Legitimacy of Targeted Welfare: Attitudes to Welfare Deservingness*, pp. 241–259.

van Doorn B and Bos A (2017) Are Visual Depictions of Poverty in the US Gendered and Racialized? In: Van Oorschot, Wim, Roosma F, Meuleman B, et al. (eds) *The Social Legitimacy of Targeted Welfare: Attitudes to Welfare Deservingness*. Cheltenham: Edward Elgar Publishing, pp. 113–126.

van Oorschot W (2000) Who should get what, and why? On deservingness criteria and the conditionality of solidarity among the public. *Policy & Politics* 28(1): 33–48.

van Oorschot W (2006) Making the difference in social Europe: deservingness perceptions among citizens of European welfare states. *Journal of European Social Policy* 16(1): 23–42.

van Oorschot W (2008) Solidarity towards immigrants in European welfare states. *International Journal of Social Welfare* 17: 3–14.
